# Atherosclerosis of the iliac arteries for the prediction of erectile dysfunction and epistaxis in men undergoing abdominal CT scan

**DOI:** 10.1186/s12894-023-01340-4

**Published:** 2023-10-27

**Authors:** Florian A Schmid, Victor Mergen, Timo Bärlocher, Basil Kaufmann, Lorenz Epprecht, Michael B. Soyka, Daniel Eberli, Andreas M Hötker

**Affiliations:** 1 Department of Urology, University Hospital Zurich, University of Zurich, Frauenklinikstrasse 10, Zurich, 8091 Switzerland; 2 Institute of Diagnostic and Interventional Radiology, University Hospital Zurich, University of Zurich, Zurich, Switzerland; 3 Department of Otorhinolaryngology, Head and Neck Surgery, University Hospital Zurich, University of Zurich, Zurich, Switzerland

**Keywords:** Calcium scoring, Agatston score, Atherosclerotic lesions, Subclinical Cardiovascular Disease, Erectile function, Nosebleed, Radiological assessment

## Abstract

**Background:**

To investigate the association between erectile dysfunction (ED) as well as epistaxis (ES) in relation to the extent of iliac atherosclerosis.

**Methods:**

In this retrospective cross-sectional study, all consecutive male patients treated at our institution from 01/2016 to 12/2020 undergoing abdominal CT scan were evaluated. Patients (n = 1272) were invited by mail to participate in the study in returning two questionnaires for the evaluation of ED (IIEF-5) and ES. Patients who returned filled-in questionnaires within a 3-month deadline were included in the study. The extent of atherosclerosis in the common iliac artery (CIA) and the internal iliac artery (IIA) was assessed by calcium scoring on unenhanced CT. Stratification of results was performed according to reported IIEF-5 scores and consequential ED groups.

**Results:**

In total, 437 patients (34.4% of contacted) met the inclusion criteria. Forty-two patients did not fulfill predefined age requirements (< 75 years) and 120 patients had to be excluded as calcium scoring on nonenhanced CT was not feasible. Finally, 275 patients were included in the analysis and stratified into groups of “no-mild” (n = 146) and “moderate-severe” (n = 129) ED. The calcium score (r=-0.28, p < 0.001) and the number of atherosclerotic lesions (r=-0.32, p < 0.001) in the CIA + IIA showed a significant negative correlation to the IIEF-5 score, respectively. Patients differed significantly in CIA + IIA calcium score (difference: 167.4, p < 0.001) and number of atherosclerotic lesions (difference: 5.00, p < 0.001) when belonging to the “no-mild” vs. “moderate-severe” ED group, respectively. A multivariable regression model, after adjusting for relevant baseline characteristics, showed that the number of atherosclerotic CIA + IIA lesions was an independent predictor of ED (OR = 1.05, p = 0.036), whereas CIA + IIA calcium score was not (OR = 1.00031, p = 0.20). No relevant correlation was found between ES episodes and IIEF-5 scores (r=-0.069, p = 0.25), CIA + IIA calcium score (r=-0.10, p = 0.87) or number of atherosclerotic CIA + IIA lesions (r=-0.032, p = 0.60), respectively.

**Conclusions:**

The number of atherosclerotic lesions in the iliac arteries on nonenhanced abdominal CT scans is associated with the severity of ED. This may be used to identify subclinical cardiovascular disease and to quantify the risk for cardiovascular hazards in the future.

**Trial registration:**

BASEC-Nr. 2020 − 01637.

**Supplementary Information:**

The online version contains supplementary material available at 10.1186/s12894-023-01340-4.

## Introduction

During the last decade, increasing evidence has demonstrated a firm association between erectile dysfunction (ED) and cardiovascular disease (CVD) as different manifestations of the same systemic disorder [1, 2]. Various studies presented evidence of ED being a marker for CVD risk and meanwhile understood ED as a clinical condition that precedes coronary artery disease [3–5]. Predominantly vascular ED is recognised as a sign of subclinical CVD [6]. CVD and sudden cardiac deaths rank among the leading causes of morbidity and mortality worldwide and are therefore acknowledged as major healthcare problems [7, 8]. The identification of early stages and the knowledge about risk factors is of tremendous importance for the prevention and prophylaxis of this condition [9, 10]. Early lifestyle modifications and pharmacological therapies can reduce the progression of CVD with its potentially deleterious consequences.

Microvascular dysfunction due to atherosclerosis stands at the origin of the pathogenic pathway of ischemic or hemorrhagic circulatory disorders [11, 12]. Next to known CVD risk scores and calculators using mainly clinical information, early vascular dysfunction can also be diagnosed by blood markers that are capable to identify patients who would benefit from a long-term monitoring with consequent prevention of major adverse cardiovascular events [13]. It is known that diseases triggered by atherosclerotic changes become manifest as microvascular dysfunction in the smallest vessels first and that patients tend to suffer later vascular complications in accordance with the increasing arterial diameter [14, 15]. Apart from the perfusion of the lower pelvis and the external genitalia, providing the blood supply for erectile function, small vessels are also found in the nasal mucosa. The friability of these small vessels is a predisposition for nosebleeds, whereas epistaxis (ES) is also known to be linked to CVD risk factors such as long-term hypertension and diabetes [16]. Therefore, ES as a symptom is thought to be associated with subclinical atherosclerosis. A recent study presented reasonable evidence for the relation of severe ES and CVD [17].

Until today, various publications have established solid evidence demonstrating that scores quantifying the extent of calcifications of the abdominal aorta – determined on nonenhanced computed tomography (CT) scans – may serve as marker for the presence of asymptomatic coronary artery disease [18–21]. The so called “Agatston Score” is a validated method for the quantification of coronary artery calcium on nonenhanced cardiac CT images and provides independent prognostic information for the prediction of future adverse cardiovascular events [22–24].

The aim of our study was to quantify the association between ED as well as ES and the extent of iliac atherosclerosis on nonenhanced abdominal CT scans in urological patients. This may help to further understand the underlying pathogenetic mechanisms of ED and ES as potential precursor sign of later CVD.

## Methods

### Patient sample

In this retrospective cross-sectional study, all consecutive male patients treated at our institution from 01/2016 to 12/2020 and undergoing an abdominal CT scan including a nonenhanced phase were evaluated. The reason of referral or the indication for performing a CT scan did not influence patient selection. Patients were contacted by mail (n = 1272) in 01/2021 and invited to participate in the study in returning a prepaid envelope with a signed patient informed consent (PIC) and two filled-in questionnaires for the evaluation of ED (International Index on Erectile Function – abridged 5-item version = IIEF-5) and ES. Patients who returned a signed PIC together with completely filled-in questionnaires within a 3-month deadline and with age ≤ 75 years were included in the study. Patients were excluded from radiological evaluation if the pelvic area was not included in the CT scan. The study was approved by the local ethics committee (BASEC-Nr. 2020 − 01637).

### Clinical parameters

Clinical parameters were gathered from the patient records. Important concurrent baseline characteristics of potential influence were identified as follows: age, body-mass index (BMI), smoking status, intake and type of oral anticoagulation, presence of diabetes mellitus (D.m.), cardio- or cerebrovascular disease (arterial hypertension, coronary artery disease, heart insufficiency, stroke [ischemic or hemorrhagic], peripheral artery disease), chronic kidney disease, prostate cancer, or presence of other carcinomas.

### Questionnaires

The IIEF-5 is a simple and standardized questionnaire to diagnose the presence and assess the severity of ED [25]. For each of the five questions, the answers resulted in scores from 0 to 1 (lowest) to 5 (highest). Based on the total summated score, patients are subdivided into groups referring to the severity, such as “no” (25 − 22 points), “mild” (21 − 17), “moderate” (16 − 12), and “severe” (11 − 1) ED. In addition, all patients were asked to indicate the use of ED aids if applied in more than 50% of sexual activities. The available options were the usage of oral PDE-5-inhibitors, intraurethral application of prostaglandin (i.e. Muse^Ⓒ^), intracavernosal injection of prostaglandin (i.e. Caverject^Ⓒ^), penile ring, or penile vacuum pump (alone or in combination).

The ES questionnaire evaluates the occurrence of ES, the number of episodes per year, duration of episodes, suspected reasons for the starting of bleeding, intensity (one or two nostrils affected), intake of oral anticoagulation, seasonal correlation, age of maximum severity, need for medical attention or transfusions, and type of intervention. Please find the ES questionnaire attached as *Appendix 1*.

### Penile arterial blood supply

The internal pudendal artery (IPA) and accessory pudendal arteries are the main source of blood supply for the penis and therefore closely linked to erectile function in men [26]. The IPA commonly evolves from the internal iliac artery (IIA), which is one of two main branches coming from the common iliac artery (CIA). The other branch is the external iliac artery (EIA), which is responsible for the blood supply of the lower extremity. Penile blood supply is therefore mainly regarded as blood flow coming from the abdominal aorta to the CIA through the IIA to the IPA. Thus, the CIA and the IIA were defined as main targets for assessing atherosclerotic lesions and performing calcium scoring on nonenhanced abdominal CT scans.

### CT data acquisition and image reconstruction

**Fig. 1:**
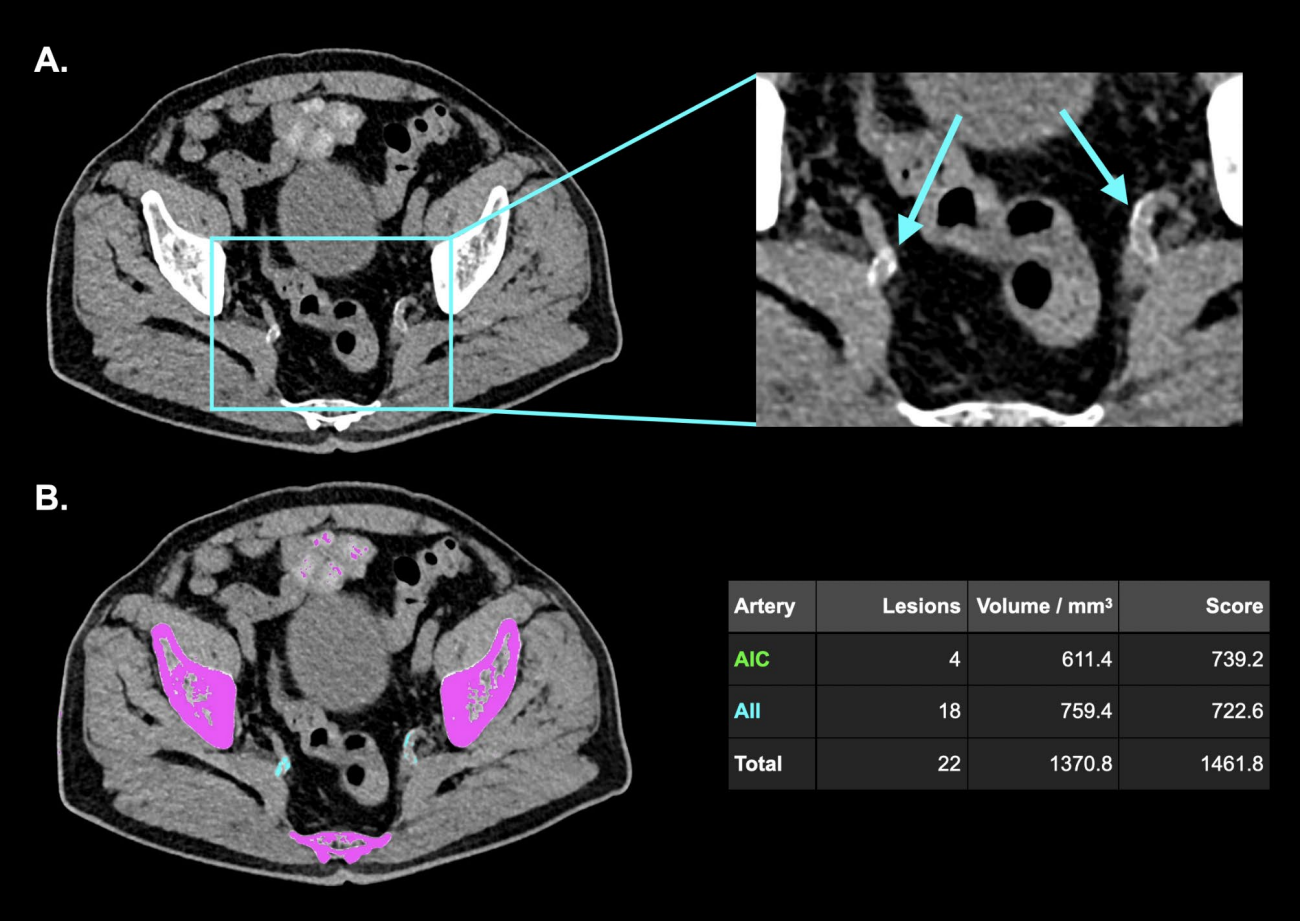
Representative CT image illustrating calcium quantification. Axial CT images of a 73-year-old male patient with calcifications of the internal iliac arteries (**A**). Calcifications are manually assigned to a corresponding artery after they have been automatically detected by the software (**B**). Calcification scores of the common iliac arteries and internal iliac arteries were 739.2 and 722.6, respectively *Abbreviations:* AIC = common iliac arteries, AII = internal iliac arteries

All patients underwent nonenhanced CT scans on a third-generation energy-integrating detector CT. Scans were acquired using automated tube voltage selection and tube current modulation. CT images were reconstructed with a slice thickness of 2 mm and an increment of 1.6 mm. Calcifications of the iliac arteries were quantified by one reader (V.M.), who was blinded to the IIEF-5 score, using a dedicated, commercially available software (CaScore, Syngo.via VB60, Siemens) in accordance with the Agatston method [24]. Output data of the software included number, volume (in mm^3^) and score for the CIA, EIA, and IIA. Figure 1 provides a representative example of the calcium scoring method.

### Statistical analysis

The statistical analysis was performed using *R* for statistical computing, version 4.0.2 [27]. Descriptive statistics are presented as absolute numbers, proportions, or percentages, and median or mean values were reported with the according standard deviation (SD). Stratification of results was performed according to reported IIEF-5 scores and their ED group. Shapiro-Wilk normality test was employed and confirmed a non-normal distribution of data. Binary, categorical, and continuous values were therefore compared using Pearson’s correlation test, Wilcoxon test and Kruskal-Wallis test. Finally, a multivariable regression analysis was fitted to predict the ED group of patients according to the calculated calcium score or the number of atherosclerotic lesions of the CIA + IIA adjusted for relevant baseline characteristics.

## Results

Four hundred thirty-seven eligible patients (34.4% of contacted patients) correctly returned the signed PICs along with the completed ED and ES questionnaires within the 3-month deadline. Forty-two patients did not fulfill predefined age requirements (age of < 75 years) and 120 patients had to be excluded as calcium scoring on nonenhanced CT was not feasible. A total of 275 patients were included in the final analysis. Baseline characteristics are shown in Table 1 stratified according to ED groups “no-mild” (IIEF-5 score: 25 − 17 points) vs. “moderate-severe” ED (IIEF-5 score: 16 − 1 points). Mean volume CT dose index (CTDI_vol_) and dose-length-product (DLP) were 6.7 mGy and 292.7 mGy $\bullet$ cm, respectively. Clinical characteristics were evenly distributed among ED groups – except of age, presence of D.m., CVD, and use of ED aids according to the attached exploratory p-values.

**Table 1:** Clinical baseline characteristics stratified according to ED groups “no-mild” vs. “moderate-severe”. Data presented as means (+/- SD) or absolute numbers (%). Exploratory p-values attached

	ED group		
	***no-mild*** *(IIEF-5: 25 − 17 points)*	***moderate-severe*** *(IIEF-5: 16 − 1 points)*	***p-value***
**n**	146	129	
**Age (SD)**	50.27 (13.11)	60.64 (11.34)	< 0.001
**BMI (SD)**	26.18 (4.75)	27.01 (4.07)	0.145
**Smoking (%)**	24 (16.4)	32 (24.8)	0.117
**OAC (%)**	9 (20.5)	20 (39.2)	0.079
**D.m. (%)**	9 (6.2)	18 (14.0)	0.050
**CVD (%)**	40 (27.4)	66 (51.2)	< 0.001
**CKD (%)**	16 (11.0)	22 (17.1)	0.198
**PCa (%)**	2 (1.4)	6 (6.5)	0.085
**other Ca (%)**	26 (17.8)	34 (26.4)	0.117
**ED aids (%)**	12 (8.6)	31 (23.0)	0.002

*Abbreviations: ED = erectile dysfunction, IIEF-5 = international index of erectile function 5 questionnaire, SD = standard deviation, BMI = body-mass index, OAC = oral anticoagulation, D.m. = diabetes mellitus, CVD = cardiovascular disease, CKD = chronic kidney disease, PCa = prostate cancer, Ca = Cancer, ED aids = usage of oral PDE-5-inhibitor, intraurethral prostaglandin, intracavernosal prostaglandin, penile ring or penile vacuum pump (alone or in combination) in > 50% of sexual activities*

### Radiological exclusions

Patients with image artifacts in the pelvis (e.g., metal artifacts from hip prostheses or with endovascular prostheses in the iliac arteries) were excluded because both problems precluded accurate calcium quantification. Moreover, the dedicated post-processing program was unable to quantify extensive confluent calcifications involving several vessels (e.g., extending from the aorta into the iliac vessels). Considering this, 120 patients had to be excluded from analysis.

### Atherosclerosis and erectile dysfunction

**Fig. 2:**
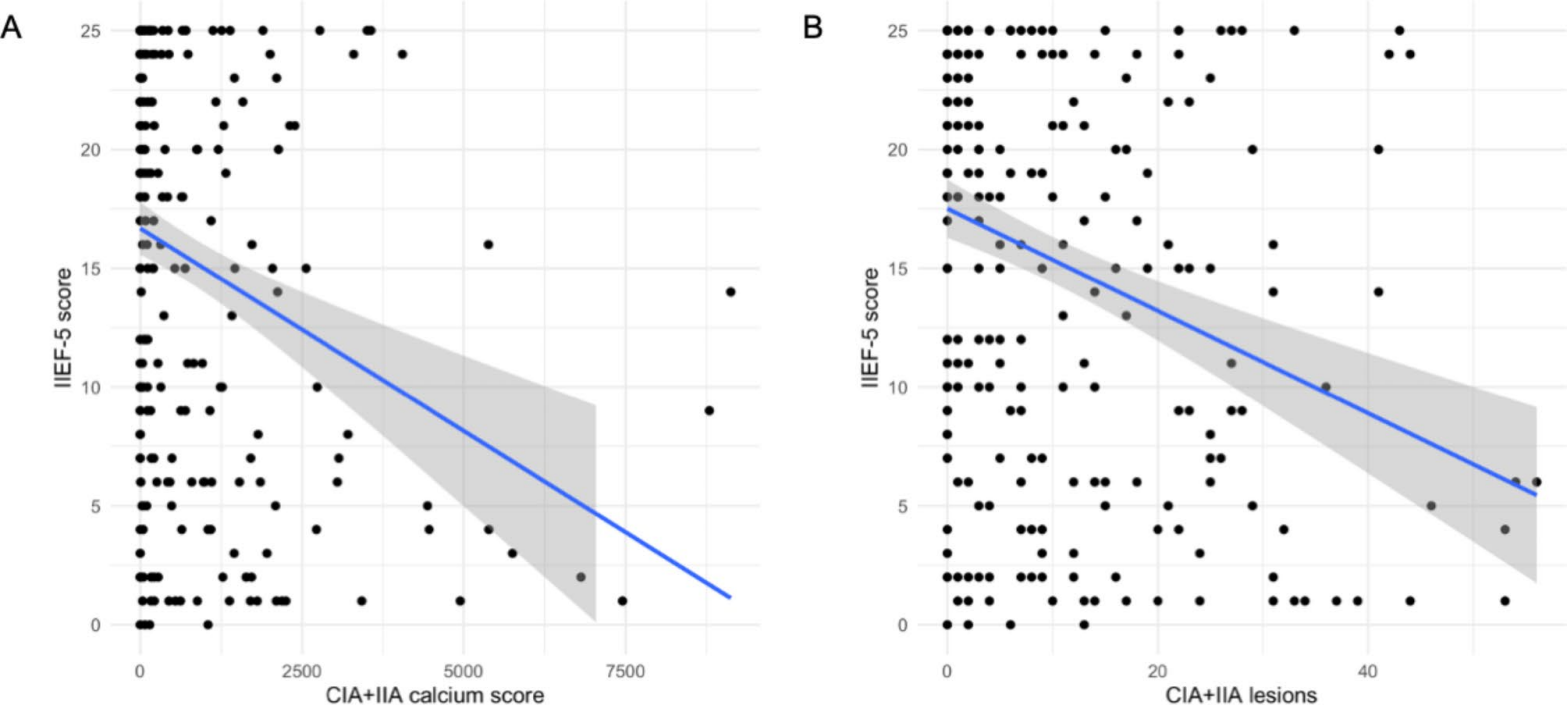
Correlation plots of IIEF-5 score and CIA + IIA calcium score (**A**) and number of atherosclerotic CIA + IIA lesions (**B**), respectively *Abbreviations:* IIEF-5 = international index of erectile function questionnaire, CIA = common iliac artery, IIA = internal iliac artery

**Fig. 3:**
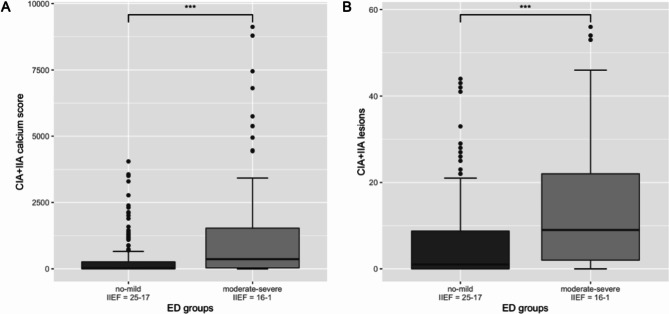
Boxplots of CIA + IIA calcium score (**A**) and number of atherosclerotic CIA + IIA lesions (**B**) according to “no-mild” vs. “moderate-severe” ED groups, respectively *Abbreviations:* CIA = common iliac artery, IIA = internal iliac artery, ED = erectile dysfunction, IIEF = international index of erectile function

**Fig. 4:**
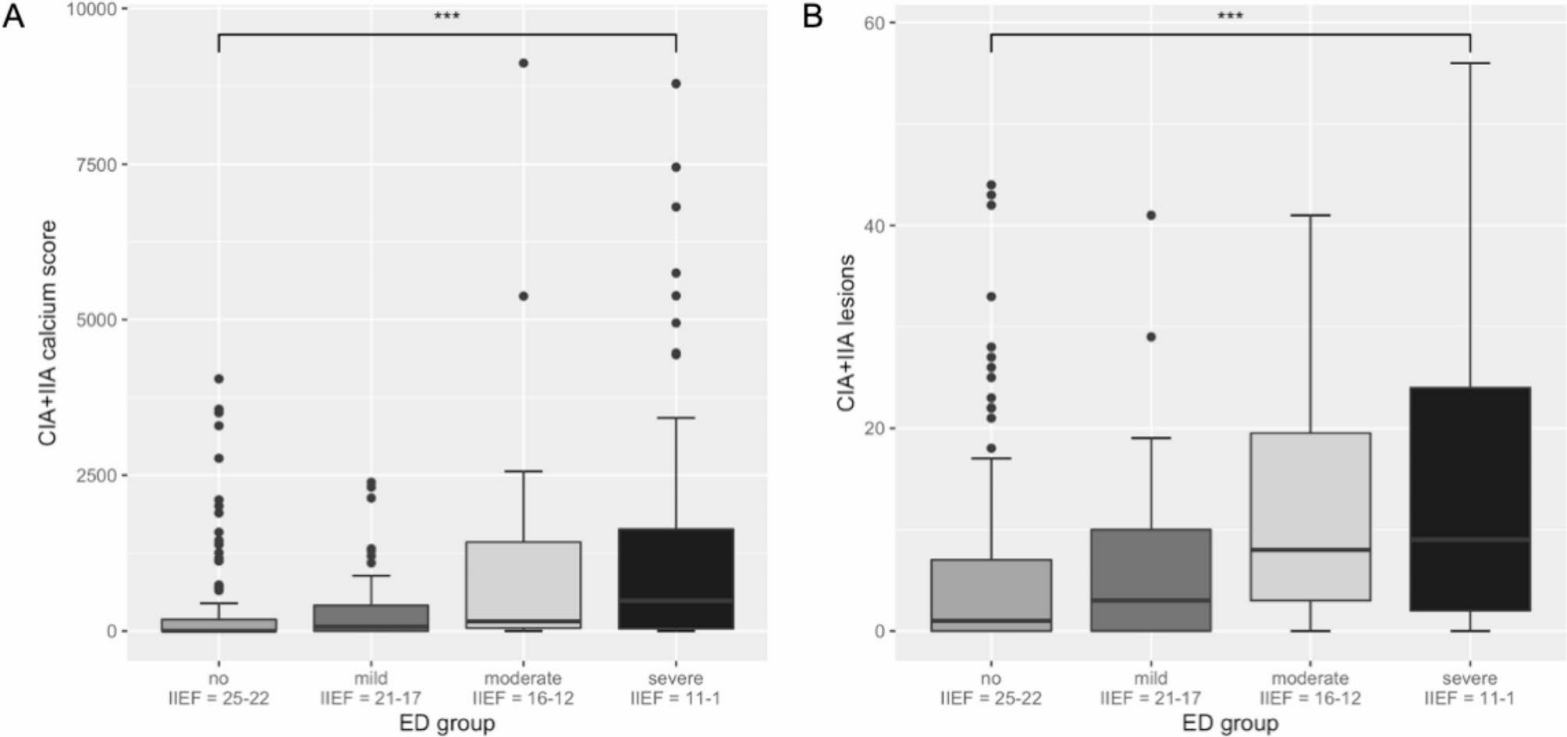
Boxplots of CIA + IIA calcium score (**A**) and number of atherosclerotic CIA + IIA lesions (**B**) according to “no”, “mild”, “moderate” and “severe” ED groups, respectively *Abbreviations:* CIA = common iliac artery, IIA = internal iliac artery, ED = erectile dysfunction, IIEF = international index of erectile function

Total calcium score as well as total number atherosclerotic lesions in the CIA + IIA showed a statistically significant negative correlation to the IIEF-5 score (r = -0.28, 95% CI: -0.38 to -0.16, p < 0.001 and r = -0.32, 95% CI: -0.42 to -0.21, p < 0.001, respectively). The according correlation plots are presented in Fig. 2. Wilcoxon test demonstrated that patients had a significantly lower CIA + IIA calcium score when belonging to the “no-mild” ED group compared to patients in the “moderate-severe” ED group (difference: 167.4, 95% CI: 68.1 to 394.4, p < 0.001). Similarly, the number of atherosclerotic lesions in the CIA + IIA differed statistically significant between the “no-mild” vs. “moderate-severe” ED group (difference: 5.00, 95% CI: 3.00 to 7.00, p < 0.001). Two boxplots present the difference between CIA + IIA calcium score as well as the number of atherosclerotic CIA + IIA lesions according to “no-mild” vs. “moderate-severe” ED groups (Fig. 3). Kruskal-Wallis test for the comparison of “no”, “mild”, “moderate” and “severe” ED groups revealed statistically significant differences in CIA + IIA calcium score (*X*^2^[3,275] = 31.7, p < 0.001) and number of atherosclerotic CIA + IIA lesions (*X*^2^[3,275] = 32.4, p < 0.001), respectively. Boxplots of CIA + IIA calcium score and atherosclerotic CIA + IIA lesions according to the 4 different ED groups are shown in Fig. 4.

### Multivariable regression analysis

The value of measuring CIA + IIA calcium score and number of atherosclerotic CIA + IIA lesions for the prediction of the according ED group (“no”, “mild”, “moderate” or “severe”) was tested in a multivariable regression model after adjusting for the relevant baseline characteristics age, BMI, smoking, D.m., and CVD. Total number of atherosclerotic CIA + IIA lesions was an independent predictor of ED (OR = 1.05, 95% CI: 1.00 to 1.11, p = 0.036), whereas CIA + IIA calcium score was not (OR = 1.00031, 95% CI: 1.00 to 1.00079, p = 0.20). Results are demonstrated in Table 2.

**Table 2a:** Multivariable regression analysis for the prediction of ED group (“no” vs. “mild” vs. “moderate” vs. “severe”) according to calculated CIA + IIA calcium score in native adominal CT scans adjusted for baseline parameters age, BMI, smoking, D.m., and CVD

ED group	OR	95%	CI	p-value
**CIA + IIA calcium score**	1.00031	1.00 -	1.00079	0.20
**Age**	1.002	0.97 -	1.036	0.91
**BMI**	0.94	0.87 -	1.012	0.097
**Smoking**	3.38	0.96 -	11.95	0.059
**D.m.**	0.66	0.21 -	2.097	0.48
**CVD**	1.4	0.59 -	3.30	0.45

**Table 2b:** Multivariable regression analysis for the prediction of ED group (“no” vs. “mild” vs. “moderate” vs. “severe”) according to the number of atherosclerotic CIA + IIA lesions in native abdominal CT scans adjusted for baseline parameters age, BMI, smoking, D.m., and CVD

ED group	OR	95%	CI	p-value
**CIA + IIA lesions**	1.054	1.0034 -	1.11	0.036*
**Age**	0.99	0.96 -	1.027	0.60
**BMI**	0.94	0.87 -	1.012	0.096
**Smoking**	3.029	0.86 -	10.69	0.085
**D.m.**	0.64	0.20 -	2.062	0.46
**CVD**	1.44	0.61 -	3.40	0.41

ED = erectile dysfunction, OR = odds ratio, 95% CI = 95% confidence interval, CIA = common iliac artery, IIA = internal iliac artery, BMI = body-mass index, D.m. = diabetes mellitus, CVD = cardiovascular disease

### Epistaxis and erectile dysfunction

**Fig. 5:**
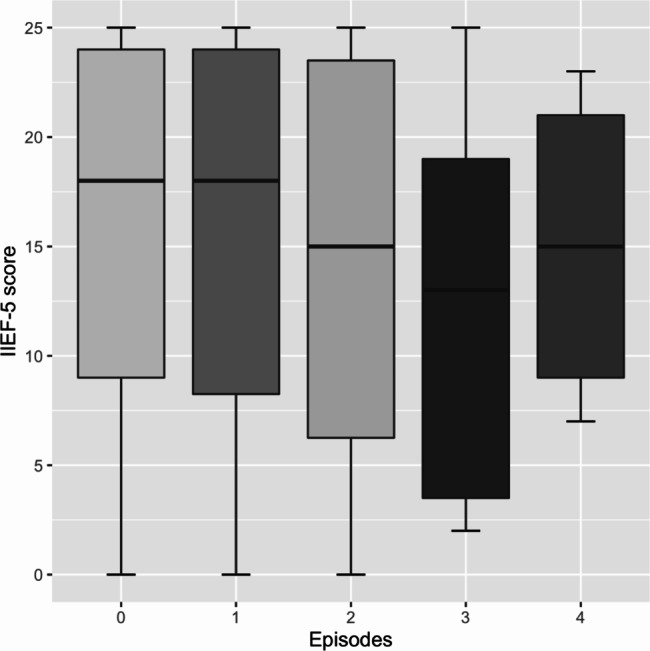
Boxplot for the comparison of IIEF-5 score in patients with ES and their according number of ES episodes *Abbreviations:* IIEF-5 = international index of erectile function questionnaire, ES = epistaxis, Episodes: 0 = none, 1 = ≤ 1x/year, 2 = 1-5x/year, 3 = 6-12x/year, 4 = > 12x/year

Pearson’s correlation test was employed to investigate the association of ES episodes and IIEF-5 scores in our patient sample and demonstrated no relevant correlation (r = -0.069, 95% CI: -0.19 to 0.050, p = 0.25). Further, no relevant correlation between ES episodes and the CIA + IIA calcium score (r = -0.10, 95% CI: -0.13 to 0.11, p = 0.87) as well as the number of atherosclerotic CIA + IIA lesions (r = -0.032, 95% CI: -0.15 to 0.087, p = 0.60) was found, respectively. Therefore, no further statistical test was used to investigate the differences between patients with ES and IIEF-5 scores or the radiological assessment of CIA + IIA atherosclerosis. However, a boxplot comparing IIEF-5 scores of patients who also experienced ES (n = 81) and their according number of ES episodes is shown as Fig. 5.

## Discussion

Our study demonstrates that the severity of ED may be predicted through the atherosclerotic burden of the iliac arteries assessed by nonenhanced abdominal CT scans. Herewith, total calcium score and total number of atherosclerotic lesions in the CIA and IIA were able to differentiate between ED groups according to IIEF-5 questionnaires. Total number of atherosclerotic lesions stayed an independent predictor of ED, after adjusting for the most relevant baseline variables such as age, BMI, smoking status, and the presence of D.m. or CVD. These findings corroborate the correlation of the clinical presence of atherosclerosis and the severity of ED on a pathophysiological level. Together with previous studies showing a correlation between the calcification of the abdominal aorta and CVD [20, 21], the findings of this study strongly suggest that ED and CVD have similar underlying pathological pathways. This is of potential importance for the screening and the early identification of subclinical CVD in patients with ED undergoing abdominal CT scans to prevent the impending clinical and health-associated consequences of circulatory disorders. Several studies have shown that ED is closely linked to, and with increasing evidence a potential precursor of CVD [3, 6]. In the sense of an ongoing atherosclerotic process, extent of CVD may be assessed on nonenhanced abdominal CT scans in a standardized manner by calcium scoring and may serve as a marker for future adverse cardiovascular events. Due to the limited number of patients with concurrent ES in our patient sample, we were not able to find a direct association of ES and concurrent ED as potential precursors of later CVD. However, a trend was identified regarding the number of ES episodes in relation to ED severity as of lower IIEF-5 scores.

The diagnostic workup in the urological patient often entails abdominal CT scans, either nonenhanced or in combination with contrast-enhanced sequences. Indications vary from the emergency setting, to elective, and oncological follow-up investigations. Especially in urolithiasis, nonenhanced abdominal CT is considered the reference standard and can be performed at a low dose without compromising diagnostic accuracy [28]. Further, CT allows for the evaluation of several differential diagnoses [29]. The quantification of aortic abdominal calcification on nonenhanced abdominal CT scans can reliably predict future cardiovascular events and has proven to outperform the Framingham risk score [21]. In addition, the abdominal aortic calcium score is an independent predictor for the presence or absence of coronary artery disease [18, 19, 30]. In combination with the assessment of clinical conditions, such as ED evaluated through standardized questionnaires, the diagnostic accuracy may be further ameliorated. To the authors knowledge, this is the first study to investigate the calcium score of the CIA and the IIA together with IIEF-5 questionnaires for the prediction of ED severity. Since ED is a known harbinger of future cardiovascular events [4, 31], our findings suggest a potential opportunistic role in abdominal nonenhanced CT scans performed for other clinical indications and should prompt investigation and intervention for cardiovascular risk factors. In addition, the use of ED aids in the 2 groups “no-mild” vs. “moderate-severe” were not evenly distributed in the underlying cohort, favoring the “moderate-severe” group. This supports our theory of the clinical relevance of iliac atherosclerosis even more, since IIEF-5 scores get better with the use of ED aids and therefore the differences between patients in the 2 ED groups become smaller than they really are.

Kunz et al. have demonstrated a close association of severe ES and the prevalence of CVD. The measurement of carotid artery intima-media thickness was on average 26% higher in patients with ES [17]. The carotid artery intima-media thickness is a well-established surrogate marker for generalized atherosclerosis and CVD [32]. In this study, we were therefore interested if there was a correlation between ED and ES indicating the same underlying pathophysiology of atherosclerosis represented by the CIA and IIA calcium score. We were, however, not able to demonstrate this relationship in our retrospective cross-sectional cohort. We were only able to show a non-significant trend of lower median IIEF-5 scores in patients with higher number of ES episodes. This means that the atherosclerotic process might affect ES and ED by a different mechanism, or that the systemic disease manifests itself in the nose at later stages. Supporting these theories are two findings from recent studies: First, ES is an independent risk factor for increased mortality [33], suggesting a more advanced atherosclerotic disease. Second, Kunz et al. studied ES patients in the emergency department, who therefore most probably had more severe ES than the retrospectively assessed population in this study who did not explicitly seek medical attention for ES and whose majority reported minor ES, only. The results of this study suggest that long-term prospective studies are needed to further investigate the relationship of ED, ES, and the time course of atherosclerotic evolution.

### Limitations

The study design of a retrospective cross-sectional study has the potential for various types of biases for which results need to be interpreted with caution. Especially the presence of selection and self-reporting bias are inherent with the underlying methodological design. In addition, because of the retrospective design of this study, unenhanced abdominal CT scans were acquired with different tube voltages, which may affect calcium quantification. Further, patients included as clinical cohort have potential confounders that are hard to be controlled for, except for known pathophysiological relations for which adjusting was feasible in the multivariable regression analysis. Lastly, a time lag of up to 4 years between performed CT scans and the receipt of ED/ES questionnaires was possible. This may influence the inference about the timely association of the atherosclerotic burden in iliac arterial branches and the severity of ED and ES. A further limitation is the exclusion of patients due to technical issues when performing calcium scoring with the dedicated software. Therefore, our findings need to be further evaluated in prospective and accordingly powered trials to investigate the details of the associated atherosclerotic process in patients with ED, ES, and subsequent CVD.

## Conclusion

The number of atherosclerotic lesions within the iliac arteries assessed by nonenhanced abdominal CT scans is associated with ED severity. This may be used to identify subclinical CVD and to quantify the risk for cardiovascular hazards in the future. Further research needs to confirm this relationship in accordingly powered and prospectively designed studies.

### Electronic supplementary material

Below is the link to the electronic supplementary material.

Supplementary Material 1

## Data Availability

The datasets used and/or analysed during the current study are available from the corresponding author on reasonable request.

## Abbreviations

BMI: body-mass index
CI: confidence interval
CIA: common iliac artery
CT: computed tomography
CTDI_vol_: CT dose index
CVD: cardiovascular disease
DLP: dose-length-product
D.m.: diabetes mellitus
EIA: external iliac artery
ED: erectile dysfunction
ES: epistaxis
IIA: internal iliac artery
IIEF-5: International Index on Erectile Function – abridged 5-item version
IPA: internal pudendal artery
OR: odds ratio
PIC: patient informed consent
SD: standard deviation
