# Supplementary material for: Atherosclerosis of the iliac arteries for the prediction of erectile dysfunction and epistaxis in men undergoing abdominal CT scan
Source: BMC Urol. 2023 Oct 27;23:173. doi: 10.1186/s12894-023-01340-4 (PMC10612309; doi:10.1186/s12894-023-01340-4)
Supplement: Supplementary file 1 — Supplementary Material 1 [file 12894_2023_1340_MOESM1_ESM.docx]

**Do you often suffer from nosebleeds?**

yes □ not relevant □ never □

**If you answered yes,** please answer the following questions:

Average presentation:

Number of episodes per year: □ less than 1x

□ 1-5x

□ 6-12x

□ more than 12x

Average duration of □ 1 minute or less

individual nosebleed □ 1-10 minutes

□ more than 10 minutes

Spontaneous nosebleed? □ yes □ no (e.g., trauma to the nose)

Both nostrils affected? □ never □ rarely □ most of the time

Blood thinning medication? □ yes □ no

If yes, which ones: □ Aspirin □ Plavix □ Xarelto (or similar) □ Marcoumar

Seasonal correlation? □ yes □ no

Age of maximum severity: □ below 14 years of age □ 14 – 45 years of age □ above 45 years of age

Termination of nosebleed by: □ spontaneous □ compression

□ medical attention necessary

If medical attention was necessary: □ Medical consultation

(Multiple selection possible) □ Cauterisation

□ Nasal packing (Gauze, nasal sponges, or similar)

□ Hospitalisation

□ Blood transfusion

Notes: ____________________

____________________

____________________

____________________
